# Convolutional Neural Network for Drowsiness Detection Using EEG Signals

**DOI:** 10.3390/s21051734

**Published:** 2021-03-03

**Authors:** Siwar Chaabene, Bassem Bouaziz, Amal Boudaya, Anita Hökelmann, Achraf Ammar, Lotfi Chaari

**Affiliations:** 1 Multimedia InfoRmation Systems and Advanced Computing Laboratory (MIRACL), University of Sfax, Sfax 3021, Tunisia; siwarchaabene@gmail.com (S.C.); Bassem.Bouaziz@isims.usf.tn (B.B.); amalboudaya71@gmail.com (A.B.); 2 Digital Research Center of Sfax, B.P. 275, Sakiet Ezzit, Sfax 3021, Tunisia; 3 Institute of Sport Science, Otto-von-Guericke University Magdeburg, 39104 Magdeburg, Germany; anita.hoekelmann@ovgu.de; 4 Interdisciplinary Laboratory in Neurosciences, Physiology and Psychology: Physical Activity, Health and Learning (LINP2), UFR STAPS, UPL, Paris Nanterre University, 92000 Nanterre, France; 5 IRIT-ENSEEIHT, University of Toulouse, 31013 Toulouse, France; lotfi.chaari@toulouse-inp.fr

**Keywords:** drowsiness detection, EEG signals, *Emotiv EPOC*^+^, deep learning, data augmentation, convolutional neural networks, classification, awake/drowsy states

## Abstract

Drowsiness detection (DD) has become a relevant area of active research in biomedical signal processing. Recently, various deep learning (DL) researches based on the EEG signals have been proposed to detect fatigue conditions. The research presented in this paper proposes an EEG classification system for DD based on DL networks. However, the proposed DD system is mainly realized into two procedures; (i) data acquisition and (ii) model analysis. For the data acquisition procedure, two key steps are considered, which are the signal collection using a wearable *Emotiv EPOC${}^{+}$* headset to record 14 channels of EEG, and the signal annotation. Furthermore, a data augmentation (DA) step has been added to the proposed system to overcome the problem of over-fitting and to improve accuracy. As regards the model analysis, a comparative study is also introduced in this paper to argue the choice of DL architecture and frameworks used in our DD system. In this sense, The proposed DD protocol makes use of a convolutional neural network (CNN) architecture implemented using the Keras library. The results showed a high accuracy value (90.42%) in drowsy/awake discrimination and revealed the efficiency of the proposed DD system compared to other research works.

## 1. Introduction

Over the past three decades, we have seen changes in driving conditions and driver safety due to the vast efforts of research studies and government agencies. According to available estimates [1], more than 1.3 million people die per year, and about 20 to 50 million people suffer non-fatal injuries due to road accidents. Drowsiness and fatigue, immediately after high speed and alcoholism [2], are the main causes of traffic injuries in many areas such as aviation [3], the military sector [4] and driving [5]. However, drowsiness detection (DD) researches [6,7] have been a subject of interest in recent years. This is now a real up to date problem in the current Covid-19 pandemic [8] where medical equipment is commonly overbooked.

Drowsiness [9] is an intermediate state between wakefulness and sleep. This state is mainly defined by heaviness in terms of reaction, changes in behavior, reflex reduction, and the difficulty of keeping the head in the frontal position of the vision field. In this regard, several means such as videos [7,10] and biomedical signals [11,12] have been targeted for DD. On the one side, the video-based applications for DD are efficient and robust against noise and lighting variations [13]. Nevertheless, the biomedical signals are the best indicators of drowsiness relative to video features, according to [14]. In this context, several biomedical signals, such as electroencephalogram (EEG) [15], electrocardiogram (ECG) [16], electromyogram (EMG) [17] and electrooculogram (EOG), have been used for various DD studies [18,19,20,21]. Among them, EEG is probably the most efficient and promising modality of DD [22,23] thanks to various existing EEG-based technologies [24]. Furthermore, this modality provides a good state of DD accuracy rate and also is more appropriate than percentage-of-eye-closure (PERCLOS) [25] indicator in the evaluation of driver drowsiness. Thanks to its high temporal resolution, portability, and inexpensive cost, the *Emotiv EPOC${}^{+}$* (https://www.emotiv.com/epoc/ (accessed on 1 June 2020) headset [26] is considered one of the most commonly used among the EEG-based technologies. The neurotechnology headset is a brain measuring data hardware that enables to record brain activity using fourteen electrodes placed on the participant’s scalp. In this paper, we focus on an EEG-based DD system using the *Emotiv EPOC${}^{+}$* headset to record brain activity by analyzing the drowsy or awake states.

Over this decade, many EEG-based research works related to machine learning (ML) [27,28,29,30] have been suggested in medical diagnosis, in particular for classification-based drowsiness detection tasks. Nevertheless, some limitations appear in ML applications such as the need for a massive dataset to train, limitation predictions in return, the need of an intermediary step for feature representation and drawing conclusions to detect anomalies.

In addition, deep learning (DL) researches [31,32] have recently shown notable progress in biomedical signal analysis especially classification-based anomaly detection. However, DL [33] is now the fastest sub-field of ML technology [34] based on the artificial neural networks (ANNs) [35]. Interestingly, DL networks offer great potential for biomedical signals analysis through the simplification of raw input signals (i.e., through various steps including feature extraction, denoising, and feature selection) and the improvement of the classification results. Various DL models have been applied to biomedical signal analysis [36] particularly for recurrent neural networks (RNNs) [37], long short-term memory (LSTM) [38], auto-encoder (AE) [39], convolutional neural networks (CNNs) [40], deep stacking networks (DSNs) [41], etc. Among them, CNNs models [42] are the most frequently used in biomedical signals classification for anomaly detection due to its high classification accuracy. In this sense, several biomedical signals-based CNNs studies [43,44,45] have been suggested for anomaly detection tasks using various architectures such as CNN, visual geometry group network (VGGNet), Residual Network (ResNet), Dense Net, Inception Net, etc. In the present study, a CNN architecture is developed to classify the drowsy or awakeness states of each participant using an *Emotiv EPOC${}^{+}$* headset.

Along with the growing success of CNNs, the interest in data augmentation (DA) quickly increased. Numerous DL research works have integrated the DA technique [46,47] in the training step in order to avoid over-fitting and improve the performance of the networks by increasing accuracy. In our work, we integrated the DA technique to improve the performance of the proposed system.

According to [48], the authors proposed an algorithm that uses features learned applying a CNN to capture various latent facial characteristics and various complex nonlinear characteristics. This system is used to warn the driver of drowsiness and to prevent traffic accidents. The trained classifier results give a classification accuracy equal to 92.33%. Likewise, in [49], the authors used an RNNs architecture to detect driver fatigue in real-time. The experimental part presents good results (92.19%). In [50], the authors propose a Complex Network-Based Broad Learning System (CNBLS) to differentiate between the fatigue and alert state using EEG signals. The experimental results showed an average accuracy of around 100%. In [51], the authors suggest the detection of driver fatigue using a single EEG signal with the AlexNet CNN model. The achieved accuracy is respectively equal to 90% and 91%. According to [52], a system composed of deep CNNs and deep residual learning with EEG signals is proposed to detect mental driver fatigue. The results showed an average accuracy reaching, respectively, to 91.788% and 92.682%. In [53], the authors proposed a system to detect driver drowsiness based on differential entropy (DE) with a novel deep convolutional neural network. The experimental results showed an accuracy equal to 96%. In [54], an EEG based prediction has been developed to transform the recorded EEG into an image liked feature map applying a CNN architecture. This approach offers a 40% detection score in the drowsy class.

The aim of our paper is to develop a new EEG-based DD system based on a CNN model. Our system is validated through individual performance assessment and comparison with other CNNs architectures used in biomedical signals analysis.

The rest of this paper is divided into four sections. In Section 2, we introduce the suggested system using the *Emotiv EPOC${}^{+}$* headset. Moreover, we introduce the methodology used for EEG data acquisition as well as the architectures used for drowsiness analysis. In Section 3, the experimental results of the proposed system are listed. A discussion is given in Section 4. Finally, conclusions and future work are drawn in Section 5.

## 2. Materials and Methods

**Figure 1:**
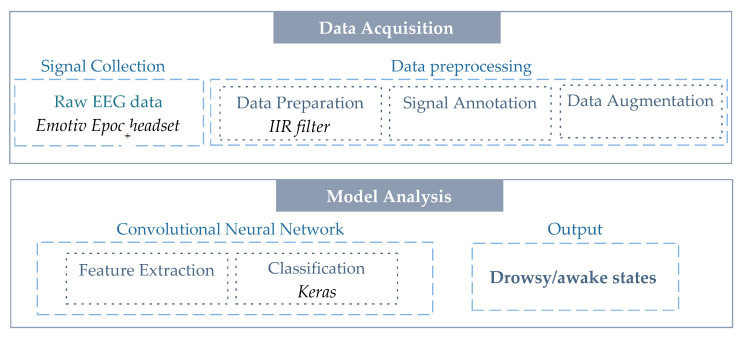
Pipeline of the proposed drowsiness detection (DD) system.

Our protocol introduces a new classification system between drowsiness or awakeness states using the *Emotiv EPOC${}^{+}$* headset to record 14 channels of EEG signals. The pipeline of the proposed system is represented in Figure 1. Data acquisition and model analysis are the two main procedures of our system. A detailed description of each procedure is given in the following subsections.

### 2.1. Data Acquisition

The EEG data acquisition procedure consists of two main steps that are signal collection using the *Emotiv EPOC${}^{+}$* headset and data preprocessing. A description of each step is provided as follows.

#### Signal Collection

**Figure 2:**
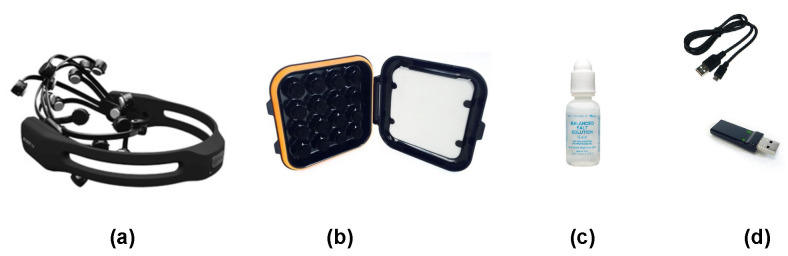
The different components of the *Emotiv EPOC${}^{+}$* headset: (**a**) helmet, (**b**) fourteen-sensors box, (**c**) saline solution and (**d**) USB Key with cable.

The signal collection step is developed by two processes, which are the hardware and the software parts [55]. The *Emotiv EPOC${}^{+}$* hardware is a non-invasive brain-computer interface (BCI) used for the development of the human brain and contextual research. Figure 2 illustrates the various *Emotiv EPOC${}^{+}$* helmet components used in the experimental step consisting of a headset, a fourteen-sensors box, a USB key with cable for battery recharging that ensures the connection between the headset and the *Emotiv Pro* software, and a saline solution [56] that ensures impedance and contact with the cortex. Compared to medical gel [57], the saline solution is easy to use and maintains effective contact with the scalp of men and women.

**Figure 3:**
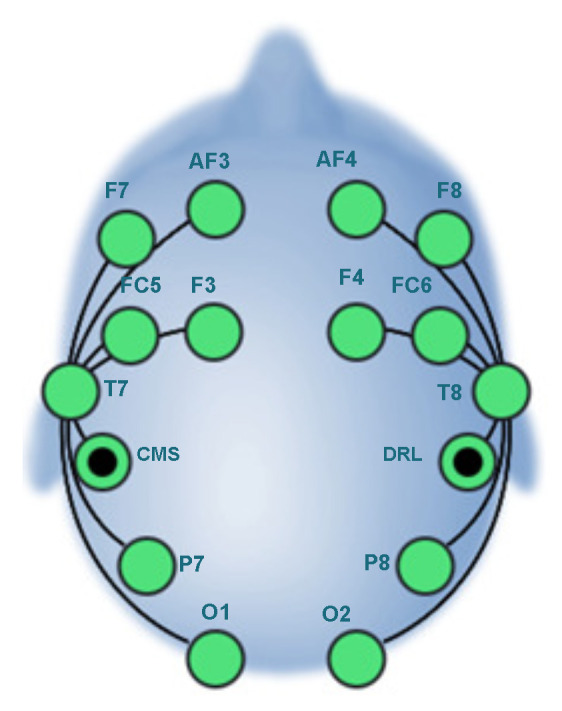
Location of the *Emotiv EPOC${}^{+}$* helmet of the International System (10–20) [58].

The *Emotiv EPOC${}^{+}$* headset provides excellent access to professional-level brain data. As shown in Figure 3, this helmet contains fourteen active electrodes with two reference electrodes, which are Driven Right Leg (DRL) and Common Mode Sense (CMS). The electrodes are mounted around the participant’s scalp in the structures of the following zones: frontal and anterior parietal (AF3, AF4, F3, F4, F7, F8, FC5, FC6), temporal (T7, T8), and occipital-parietal (O1, O2, P7, P8). Table 1 presents some of the main characteristics of the *Emotiv EPOC${}^{+}$* helmet.

The *EmotivPRO* software allows visualizing the data streams in real-time including all data sources. This program configures the vertical scaling of the EEG Graphics with the multi-channel and single-channel display mode. Subsequently, the raw EEG data are exported in European Data Format (EDF) or Comma-Separated Values (CSV) formats that are considered as the input of the data preprocessing step.

### 2.2. Data Preprocessing

The specific preprocessing steps of the EEG data revolve around the following points that are data preparation, signals annotation, and data augmentation.

#### 2.2.1. Data Preparation

Various noise sources are targeted in the portion of the raw signal including eye blinks [59,60], dipolar size variance, muscle switches, inherent electrical properties and physical arrangement of various tissues [61]. Data preprocessing is a preliminary step to EEG data denoising. In this context, various filters based on EEG denoising methods have been suggested as infinite impulse response (IIR) and finite impulse response (FIR) filters. Other sophisticated denoising approaches could be considered at the expense of higher computational complexity [62,63]. In our work, we propose to use an IIR filter that manages an impulsive signal within time and frequency domains. The IIR filter function can be expressed as the following discrete difference:

(1) $$y\left(n\right)=\sum_{m=0}^{M}b_{m}x\left(n-m\right)-\sum_{m=1}^{N}a_{m}y\left(n-m\right),$$

where *y*(*n*) refers to the filtered signal, *x*(*n*) represents the input signal, $b_{m}$ and $a_{m}$ refer to the coefficients of the filter, and N represents the order of the filter. Subsequently, we convert the EEG signal from the time domain to the frequency domain using the fast Fourier transform (FFT) [64]. The key task of the FFT is to take to 1024 samples from the input signal in the time domain and generate the output frequency of 128 Hz in the spectrum domain. In this work, alpha and theta waves analysis are accomplished using the FFT by adopting standardized EEG data.

#### 2.2.2. Signals Annotation

The central nervous system (CNS) [65] consists of the spinal cord, the cerebellum, and the brain. The latter is divided into two parts: the right and left hemispheres. There are four lobes in each hemisphere, which are frontal, parietal, occipital, and temporal. Predominantly, the EEG signal is split into large spectral frequency bands related to EEG processors and rhythms of various frequency waves [66,67]. Brainwaves are usually classified into five frequency and amplitude bands [66] including Gamma, Beta, Alpha, Theta, and Delta where each band wave refers to identifying states of participants. Other mixed bands, such as Alpha-Theta (5–9 Hz) [68], have also appeared, which refers to waking and relaxation states. Table 2 presents a brief description of each brainwave from EEG signals.

The main functions associated with the six brainwave frequencies are described in the following in order to identify the electrodes that allow the detection of drowsy/awake states.

- Gamma bands have a frequency ranging from 30 to 70 Hz and an amplitude value between 3 µV to 5 µV. These waves are used to detect Alzheimer’s disease [69].
- Beta wave is generated from the cortex region with frequency values from 13 to 30 Hz and a low amplitude ranging from 2 to 20 µV. These waves are related to awake states and various pathologies and symptoms of drugs.
- Alpha band is produced from the thalamus area with a frequency ranging between 8 to 13 Hz and amplitude values between 20 to 60 µV. This band is detected with eyes closed to generating relaxation and awake states with attenuating drowsiness.
- Theta wave is produced from the neocortex and hippocampus areas of the brain with frequency values from 4 to 7 Hz and an amplitude ranging from 20 to 100 µV. This band is correlated with a drowsiness state.
- Delta wave is produced from the thalamus with a spectrum range of 4 Hz and an amplitude ranging from 20 to 200 µV. The wave is shown in the deep stage of sleep.
- Alpha-Theta waves have a frequency ranging from 5 to 9 Hz and amplitude values between 20 to 100 µV. These bands refer to awake and drowsy states.

**Figure 4:**
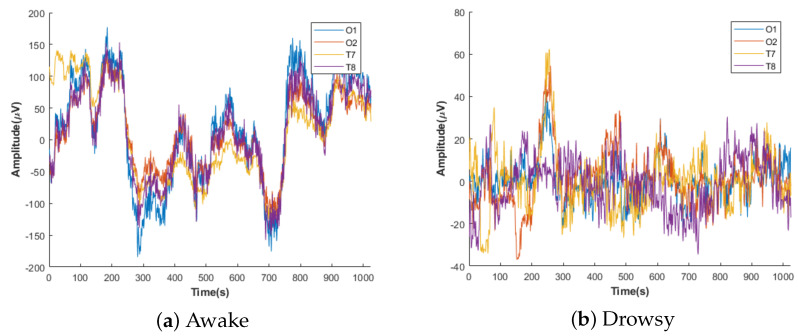
Example annotation of drowsy (**a**) and awake (**b**) of our electroencephalogram (EEG) signal collection.

Furthermore, drowsiness is an intermediate state between awakeness (i.e., wakefulness) to sleep. During awakeness, *beta* waves are analyzed in the human brain [70]. The drowsy stage is called stage 1 of sleep, the correlation is assured by *alpha* and *theta* bands [71,72,73,74]. The decrease in the *alpha* band and the rise in the *theta* frequency band expresses drowsiness [75]. The drowsy state is a transitional phase between wakefulness and sleep, which is experienced in theta brain waves. This step is characterized by a decrease in the EEG waves frequency with an increase in their amplitude. The third and fourth steps are related to deep sleep, which is characterized by a low frequency and high amplitude fluctuation of the delta waves [76]. According to this analysis, we support that the alpha-theta waves are the best bands for detecting the drowsy state. Our annotation is based on the study of *Alpha-Theta* waves for drowsiness/awakeness detection from, respectively, the occipital and temporal regions. The illustration of our annotation for the awake and drowsy states mentioned by O1, O2, T7, and T8 is shown in Figure 4. During the awakeness state, the amplitude is characterized by the lowest value while the drowsiness state is characterized by the highest value.

#### 2.2.3. Data Augmentation

In the recent year, DA [77] has been shown to achieve significant performance for DL with increasing accuracy and stability and reducing over-fitting. As developed in [46], DA is a process in which new data are artificially created from the current data on the training phase. In [78], the need for developing a DA technique contributes to avoiding over-fitting, improves classification accuracy and stability [47,79] then better generalizes on new data and enhances performance in imbalanced class issues [80]. Furthermore, DA allows improving the efficiency of CNN in the BCI field by overcoming the problems of small datasets. DA effectiveness varied considerably across techniques. However, sampling methods, noise addition, windows sliding, and Fourier transform are considered as the classic examples in signal classification tasks. Growingly, the DA techniques are used with DL networks on EEG signals works to generate new samples based on existing training data [46]. This technique presents various advantages as it increases the model robustness against the variability of the input without decreasing the efficient capacity [81]. In our work, DA steps are applied only to the training set in order to prevent over-fitting. The main idea of this procedure is to generate new samples by labeling retraining data transformations. The proposed DA method is considered as the opposite operation to dropout [82] where a small volume of training data are duplicated randomly and appended to the training set. For instance, each EEG segment of the training set added a form of opposite operation to the dropout where the segments were extended by duplicating the vectors at random time points to a fixed length in the time dimension.

### 2.3. Model Analysis

Choices of the different architectures and frameworks of DL used in the proposed DD system are argued by a comparative study. This section introduces our DD system based on comparative analysis.

#### 2.3.1. Comparative Study

Simple CNN, ResNet, WaveNet, and Inception are among the best CNNs networks widely used in biomedical signals analysis studies. Based on recent works [42,83,84,85,86,87,88,89,90,91,92,93,94,95,96,97], a comparative analysis is provided in the following using various performance criteria as *complexity*, *1D-dimension*, *performance* and *time-consumption*. In this regard, specific three tests (2, 3 and 4 states) with various values are given for each criterion as following.

- 2 states (0, 1),
- 3 states (0, 0.5, 1),
- 4 states (0, 0.33, 0.66, 1),

where 0 value is the low level, 1 value represents the high level, 0.33, 0.5, and 0.66 are intermediate levels. Table 3 indicates the score of the architectures with 2, 3, and 4 states.

For instance, 0 value indicates more *complexity* and *time-consumption*, low *performance* and unused for *1D-dimension*, while a value of 1 indicates less *complexity* and *time-consumption*, high *performance* and widely used for *1D-dimension*. The highest score is identified by the best architecture used in biomedical signals classification. According to the reported results, the high total value is presented by the Simple CNN architecture.

As regards the choice of the DL framework, there are numerous open-source frameworks [98,99], such as keras [100], tensorflow [101], and pytorch [102]. In the developing of DL models, the Keras framework offers a high level in build blocks by using particular libraries, such as TensorFlow, dedicated for operations characterized by a low level [103]. In this context, we have used the Keras DL library with a sequential model applied to the binary classification. Keras is used to build the architectures with TensorFlow backend [104]. This framework presents high-level application programming interfaces (APIs) developed on top of TensorFlow. This model is characterized by its easy use and its simplicity.

Regarding the choice of the optimization algorithm, many optimizers exist in the literature such as Adam [105], Stochastic Gradient Descent Optimizer (SGD) [106] and Root Mean Square Propagation (RMS prop) [107]. In this context, SGD is the most popular optimizer, which is simple and effective for finding optimal values in a neural network. In this work, we have used an SGD optimizer.

#### 2.3.2. Proposed Simple CNN Model

**Figure 5:**
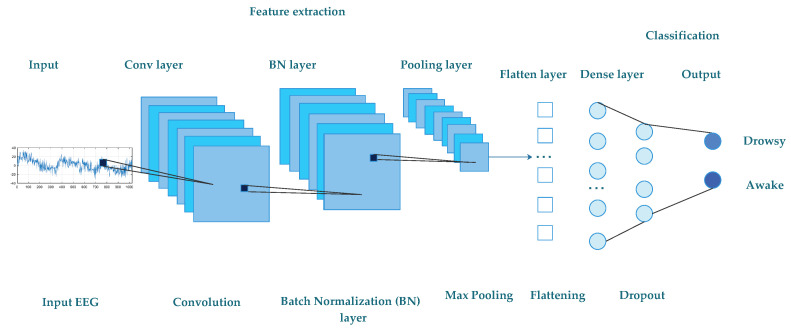
Diagram of the proposed convolutional neural network (CNN) model.

The diagram of the proposed CNN used in our DD system is presented in Figure 5. All the EEG windows with 3.75 s are the input of our proposed model. Via four convolutional and one max-pooling layers, EEG signals move followed by seven batch-normalization and one fully connected layer. All layers are equipped with the activation function of the rectified linear unit (ReLU). The pooling process chooses the maximum pooling procedure that can accomplish both reduction of dimensionality and invariance. In addition, dropout processing [82] is used to reduce the risk of over-fitting. Throughout the structure of our network, the fully connected layer serves as a classifier when mapping between high and low dimensions. The different layers of the proposed CNN model used in our DD system are detailed in the following.

- **Convolutional layers**
  The layers allow filter application and features extraction [108] based on the input EEG signals. The equation below presents the convolution operation.
  (2) $$Y_{i}=b_{i}+\sum_{n}W_{in}*X_{n},$$
  where ∗ is the convolution operation, $Y_{i}$ presents the feature map, $b_{i}$ is the bias term, $W_{in}$ is the sub-kernel of channel and $X_{n}$ is the input signal. Table 4 presents a description of the four convolutional layers purpose.
- **BatchNormalization layers**
  As known in DL, there are two fundamental problems [109], which are the over-fitting and the long training duration. The Batch Normalization (BN) layers are used to scale and speed up the learning process. Accordingly, each BN stratum normalizes the previous activation layer by subtracting the average batches, as well as divides it by the standard deviation.
- **Dropout layer**
  Each dropout layer is considered as a regularization technique and allows to improve over-adjustment on neural networks in which it decreases the error rate in the classification process. In the proposed model, the value of dropout is equal to 0.2. To avoid over-fitting, we have inactivated 20% of the neurons. We have used three dropout layers in our model.
- **Max-Pooling1D layer**
  The sample-based discretization max-pooling-1D blocks is used to sub-sample each input layer by reducing its dimensionality and decreasing the number of the parameters to learn, thereby reducing calculation costs.
- **Flatten layer**
  A multidimensional data output is given in the previous step, which cannot be read directly from this neural network, and the model is therefore flattened.
- **Dense layers**
  The dense layer has the role of describing the connectivity with the next and intermediate layers of neurons. We have used two fully connected layers in our architecture. In the first dense of our model, we used a hidden layer of 128 neurons to have better classification results. For the second dense, the value of the final neuron is equal to 1. Binary classification is applied in this work, so a single neuron is sufficient to denote class “1” or “0”.

## 3. Experimental Validation

A description of our dataset and experiments without and with DA were provided in the following subsections for the efficiency assessment of the proposed DD scheme. Our experiments have been performed using the power of GPU (Graphical Processing Unit) provided by the Google AI (Artificial Intelligence) platform and Colab [110].

### 3.1. Dataset

Our EEG signal collection contains forty-two records of six men and eight women aged between fourteen and sixty-four with normal mental health. For each person, we made three recordings lasting sixteen minutes over the day: in the morning, afternoon, and evening. For each recording, the total number of rows of data is equal to 123,648. In order to identify the state of each participant, we divided the EEG signal into windows of 3.75 s. In this sense, we split each EEG recording into 256 different sets (segments) and the length of each segment is equal to 483. Based on the proposed data annotation step in our method, a deeper analysis of the brain is the preliminary phase in the detection of each participant’s state. In this regard, we categorized the different participants according to drowsy and awake states. Table 5 presents the detailed results for each participating state.

Our data are divided into two parts, with 80% and 20%, respectively, as training for the train model and testing for the predict model. There were (20,286, 256) recordings in total in which (16,422, 256) were used for training and (3864, 256) for testing. Therefore, the training set data is divided into two parts, with 80% and 20% as training and validation. There were (16,422, 256) recording in total in which (13,137, 256) were used for training and (3285, 256) for validation.

### 3.2. Experimental Details

The different parameters as filters, kernel-size, padding, kernel-initializer, and activation of the four convolutional layers have the same values, respectively, 512, 32, same, normal, and relu. The parameter values of the remaining layers are detailed in Table 6.

We aim to reach the best accuracy rate by using a minimum number of electrodes that provide information about the drowsiness state. In [111,112,113], the authors discover that the pre-frontal and occipital cortex are the most important channel to better diagnose the drowsiness state. Furthermore, previous work [114] indicates that occipital, parietal, central and frontal regions are useful for drowsiness detection. According to the recent related work [115], the authors provide that the frontal, occipital and parietal are the best selected areas for DD. To select the relevant channels that enable the best accuracy in the proposed DD system, we suggest comparing the different results recorded by various numbers of electrodes. To reach the converge of our model, we used 15 epochs for all experiments. To this regard, we choose the following recorded data:

- Recording by 14 electrodes including the frontal and the anterior parietal (AF3, AF4, F3, F4, F7, F8, FC5, FC6), the temporal (T7, T8), and the occipital-parietal (O1, O2, P7, P8).
- Recording by 7 (AF3, F7, F3, T7, O2, P8, F8) electrodes from parietal, occipital, pre-frontal and temporal areas.
- Recording by 4 (T7,T8, O1 and O2) electrodes from the temporal and occipital areas.
- Recording by 2 (O1 and O2) electrodes from the occipital area.

#### 3.2.1. Experiments without DA

Table 7 presents the reported testing and training accuracies, respectively, with two, four, seven, and fourteen electrodes. From the reported results, the different accuracy values related to the training and validation sets as well as testing sets are low. One can notice that the training accuracy is quite stable over different electrode configurations, while test accuracy presents more disparity and lower values. These high classification error rates on the testing set indicate low generalization capacity of the proposed model when used without DA.

In the next experiments, a DA step is added to the training set to improve the classification performance (accuracy) of the proposed DD system, thereafter to select the most efficient number of electrodes associated with the best results.

#### 3.2.2. Experiments with DA

In the present work, we solve the data limitation problem by adding the DA step to increase the performance of the proposed CNN model. The DA step is only processed for the training set by using 20 duplicates. In this regard, the vector value of the training set is doubling from (13,524, 256) to (132,058, 256). The reported training, validation and testing accuracies, respectively, with two, four, seven, and fourteen electrodes are presented in Table 8. We can notice that DA allows to drastically improve the performance of the proposed model while used with seven electrodes, especially for the testing set. As regards training, the four configurations perform similarly with very good accuracies.

After evaluating our model with the use of the DA technique, we can select the best acquisition configuration, i.e., seven electrodes. To this regard, we use AF3, F7, F3 and F8 electrodes from the frontal, T7 the temporal, O2 the occipital and P8 the parietal areas. The values mentioned in Table 8 present the average accuracies of three runs for each experiment. Table 9 gives an example of the average accuracy of seven electrodes with DA.

**Figure 6:**
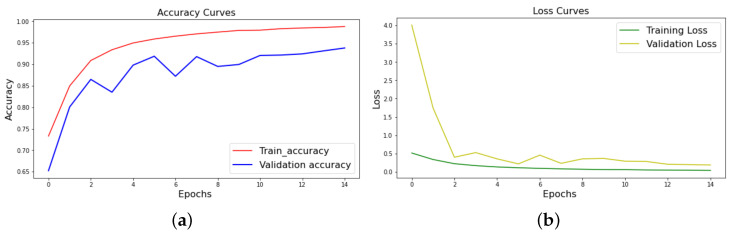
(**a**) Accuracy graph, (**b**) loss graph.

Using the selected electrodes, Figure 6 displays the training and validation accuracy and loss. Using 15 epochs, we find that the train and validation accuracy improves, and the training and validation loss decreases. This shows that the proposed CNN-based DD system has been trained to achieve up to 98.81% highest training accuracy with 90.42% highest testing accuracy for the prediction in order to automatically classify the EEG signals in drowsy/awake states.

To further quantitatively evaluate the performance of the proposed model, True Positive (TP), True Negative (TN), False Positive (FP), and False Negative (FN) rates are used to evaluate metrics [116] such as accuracy, precision, recall, and F1 score calculated as follows:

(3) Accuracy=(TP+TN)/(TP+TN+FP+FN).

(4) Precision=TP/(TP+FP).

(5) Recall=TP/(TP+FN).

(6) F1score=(Precision∗Recall)/(Precision+Recall).

In the experimental configuration with DA, the highest accuracy value is equal to 90.42%, the precision is equal to 86.51%, the recall value is equal to 89%, while the F1-score value is equal to 88%. This high precision rate indicates the capacity of the model to not miss drowsy alarms.

**Figure 7:**
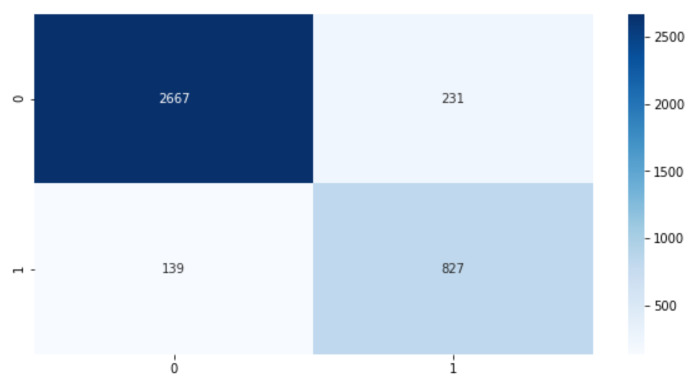
The highest results of the confusion matrix of 7 electrodes with DA.

To visualize the performance of the proposed model, we used the confusion matrix that is represented in Figure 7, where 2667 presents the TP, 231 presents the FP, 139 presents the FN and 827 presents the TN.

Additionally, the cross validation method is used in order to evaluate our model with seven electrodes. Table 10 presents all the experimental results with different folds.

### 3.3. Comparison

In order to evaluate the effectiveness of the proposed CNN model, we compared the performance measures of our model with that of several different CNNs architectures, as mentioned in Table 11, such as Inception (Conv1d_3, Conv1d_4, Conv1d_5, Max_Pooling1d_1, Concatenate_1, Batch_Normalization, Dropout, Flatten, Dense, Batch_Normalization and Dense_), WaveNet (import WaveNet) and ResNet (Conv1d_46, Conv1d_47, Conv1d_45, Add_14, Activation_14, Batchnormalization_14, Dropout_7, Flatten_5, Dense_17, Batchnormalization_15 and Dense_18).

Additionally, we compare our work with recent DD systems in the literature. In [54], the authors propose a system based on the EEG signal processing image, which converts the EEG signal into an image-like signal 2-D function map and then transfers them to the CNN model for DD. This architecture is composed of two convolutional and pooling layers with one fully connected layer. The total accuracy in the prediction imbalanced dataset result is equal to 71.15%. In [40], the authors suggest a DD system based on a DL model. Using spectrograms from the channels of EEG signals, the proposed system is developed to the ULg Multimodality Drowsiness Database. The used ConVNets model is composed of three convolutional and max-pooling layers with one fully connected layer. An accuracy of 86% is achieved in this work. We implement these two DL architectures using our EEG data. Table 12 indicates the accuracy values of the testing set using the competing DD systems. It is noteworthy that the proposed DD system gives the best accuracy classification of drowsy/awake states.

## 4. Discussion

EEG data are being increasingly used to analyze drowsiness through the control of mental states, fatigue progression, and tiredness over time [117]. Interestingly, reported studies in the literature indicate a specific trend to reduce the number of used electrodes [118,119]. From a practical point of view, reducing the number of electrodes ensures better comfort for the driver. In this paper, we started by using fourteen electrodes and we reduced the number to seven, four, and two electrodes. However, brain regions, such as the parietal, frontal, and occipital lobes, tend to be more vulnerable than other areas for DD. To this regard, alpha and theta waves from the occipital and the temporal area reveal a high indicator for DD. During drowsiness, exhaustion, and insufficient attention, the alpha band demonstrates an increase in-band power, while the theta band indicates the state of deep relaxation during the first phase of slow sleep. In fact, these waves reflect the state between sleep and wholeness. Therefore, comparative behavioral testing of alpha and theta waves can be beneficial for effective DD. The proposed DD system is divided into two steps as data acquisition and model analysis. The first step contains three steps, signal collection, data annotation, and data augmentation (DA). An *Emotiv EPOC${}^{+}$* headset is used for signal collection. Subsequently, we have annotated our dataset according to the amplitudes of alpha and theta waves. By incorporating the DA step to improve performance, we have done two experimental tests: with and without DA. For model analysis, we have built a CNN model in which implementation is done using the Keras framework. The average values of the accuracy, F1-score, precision, and recall showed a high classification rate using seven electrodes, in comparison to other competing methods.

## 5. Conclusions and Future Work

This paper proposes a new DD system based on EEG signals using a CNN architecture. An *Emotiv EPOC${}^{+}$* headset is used for signal collection. Furthermore, our EEG data has been annotated to detect drowsiness based on the analysis of alpha and theta waves from the occipital and temporal area. A study has been conducted to select the most suitable number of electrodes. Obtained results are coherent with the state-of-the-art. In this context, we proposed a system for DD using only seven electrodes. The proposed system achieves an average classification accuracy of 90.14%. In future work, EEG can be considered with other physiological assessment tools, such as EOG, ECG and Near-Infrared Spectroscopy (NIRS) [120,121], which help to improve accuracy rate. We will also consider validating our system on larger datasets, especially collected under real driving conditions.

## Figures and Tables

**Table 1:** The characteristics of the *Emotiv EPOC${}^{+}$* helmet.

Characteristics	EEG Headset
Number of channels	14 (plus 2 references CMS and DRL)
Channel names	AF3, F7, F3, FC5, T7, P7, O1, O2, P8, T8, FC6, F4, F8, AF4
Sampling rate	128 SPS (2048 Hz internal)
Sampling method	Sequential sampling
Bandwidth	0.2–45 Hz, Digital notch filters at 50 Hz and 60 Hz
Resolution	14 bits
Filtration	Sinc filter
Dynamic range	8400 µV (microvolts)

**Table 2:** Characteristics of brain waves.

Brainwaves	Description	Frequency Interval	Location
Gamma	Refers to hyper-vigilance state	>30 Hz	—
Beta	Refers to alert state	13 to 30 Hz	Frontal and Central
Alpha	Refers to waking state	8 to 13 Hz	Frontal and Occipital
Theta	Refers to the half-sleep	4 to 7 Hz	Temporal and Median
Alpha-Theta	Refers to waking and relaxation states	5 to 9 Hz	Temporal and Occipital
Delta	Refers to consciousness and sleep states	0.5 to 4 Hz	Frontal lobe

**Table 3:** The architectures scores with 2, 3, and 4 states.

CNNs Architectures	ResNet			Inception			WaveNet			Simple CNN		
References	[86,87,88,89,90]			[96,97]			[84,91]			[42,92,93,94,95]		
States number	2	3	4	2	3	4	2	3	4	2	3	4
*Complexity*	0	0	0.33	0	0	0.33	0	0	0.33	1	1	1
*Performance*	1	1	0.66	0	0.33	0.5	1	0.5	0.66	1	1	1
*Time-consumption*	0	0.5	0.66	0	0.5	0.66	1	0.5	0.66	1	0.5	0.66
*1D-dimension*	1	1	0.66	0	0.5	0.66	1	1	1	1	1	1
Total	2	2.5	2.31	0	1.33	2.15	3	2	2.65	4	3.5	3.66

**Table 4:** Convolutional layers parameters.

Parameters	Role
Filters	Feature extraction
Kernel size	Convolutional window specification
Kernel initializer	Initialization of all values
Activation	Applied after performing the convolution

**Table 5:** Detailed table of each participant’s status.

Participants	Morning	Afternoon	Evening
P1 (26 years)	Drowsy	Drowsy	Drowsy
P2 (46 years)	Awake	Drowsy	Awake
P3 (37 years)	Drowsy	Drowsy	Drowsy
P4 (35 years)	Drowsy	Drowsy	Drowsy
P5 (64 years)	Drowsy	Drowsy	Awake
P6 (62 years)	Awake	Drowsy	Drowsy
P7 (53 years)	Drowsy	Drowsy	Drowsy
P8 (63 years)	Awake	Awake	Awake
P9 (59 years)	Drowsy	Awake	Awake
P10 (24 years)	Drowsy	Drowsy	Awake
P11 (17 years)	Drowsy	Awake	Drowsy
P12 (22 years)	Drowsy	Drowsy	Drowsy
P13 (14 years)	Drowsy	Drowsy	Drowsy
P14 (43 years)	Awake	Awake	Drowsy

**Table 6:** Summary of our model.

Participants	Morning	Afternoon	Evening
Layer Num	Type	Output Shape	Parameters
Layer 1	Batch Normalization	(None, 256, 2)	1024
Layer 2	Conv 1D	(None, 256, 512)	33,280
Layer 3	Conv 1D	(None, 256, 512)	8,389,120
Layer 4	Batch Normalization	(None, 256, 512)	2048
Layer 5	Dropout	(None, 256, 512)	0
Layer 6	Conv 1DN	(None, 256, 256)	4,194,560
Layer 7	Batch Normalization	(None, 256, 256)	1024
Layer 8	Dropout	(None, 256, 256)	0
Layer 9	Batch Normalization	(None, 256, 256)	1024
Layer 10	Conv 1D	(None, 256, 256)	2,097,408
Layer 11	Batch Normalization	(None, 256, 256)	1024
Layer 12	Maxpool 1D	(None, 2, 256)	0
Layer 13	Dropout	(None, 2, 256)	0
Layer 14	Flatten	(None, 512)	0
Layer 15	Dense	(None, 128)	65,664
Layer 16	Batch Normalization	(None,128)	512
Layer 17	Dropout	(None, 128)	0
Layer 18	Batch Normalization	(None, 128)	512
Layer 19	Dense	(None, 1)	129

**Table 7:** Training, validation and testing accuracy of the various numbers of electrodes without data augmentation (DA).

Number of Electrods	2	4	7	14
Accuracy train	78.20%	85.82%	88.22%	90.46%
Accuracy Validation	74.33%	80.09%	86.30%	87.95%
Accuracy test	68.79%	54.14%	72.41%	79.43%

**Table 8:** Training, validation and testing accuracy of the various numbers of electrodes with DA.

Number of Electrods	2	4	7	14
Accuracy train	94.30%	97.25%	98.88%	93.69%
Accuracy Validation	78.14%	86.06%	93.27%	89.22%
Accuracy test	77.41%	78.49%	90.14%	82.07%

**Table 9:** Average accuracies of training, validation and testing of 7 electrodes with DA.

Run	1	2	3	Average Accuracy
Accuracy train	98.94%	98.90%	98.81 %	98.88%
Accuracy Validation	92.15%	93.88%	93.79%	93.27%
Accuracy test	90.01%	90%	90.42%	90.14%

**Table 10:** The experimental results of cross-validation for 7 electrodes with DA.

Train and Validation Sets	80%, 20%	60%, 40%	40%, 60%	20%, 80%
Accuracy train	98.94%	98.81 %	98.66%	98.83%
Accuracy Validation	92.15%	89.82%	88.32%	89.48%
Accuracy test	90.01%	88.20%	84.94%	84.96%

**Table 11:** Accuracy comparison of the proposed CNN model with ResNet, Inception and WaveNet models.

Models	Proposed CNN	Inception	Resnet	Wavenet
Accuracy train	98.88%	88.91%	79.03%	71.54%
Accuracy Validation	93.27%	67.70%	69.86%	67.40%
Accuracy test	90.14%	74.87%	72.80%	75%

**Table 12:** Accuracy test comparison with related works.

DD Methodology	Accuracy Test	Classification Method
E.J. Cheng et al. [54]	74.95%	CNN
L. Guarda et al. [40]	83.93%	ConvNets
Proposed Method	90.14%	CNN

## Data Availability

Data are available from the authors (S.C., B.B., or A.B.) upon reasonable request. Dataset: https://github.com/bassem-bouaziz/Drowsiness_Detection (accessed on 1 June 2020).

## Abbreviations

The following abbreviations are used in this manuscript:

ANNs	Artificial Neural Networks
AE	Auto-encoder
APIs	Application Programming Interfaces
AI	Artificial Intelligence
BCI	Brain Computer Interface
BN	BatchNormalization
CNN	Convolutional Neural Network
CNBLS	Complex Network-based Broad Learning System
CMS	Common Mode Sense
CSV	Comma Separated Values
CNS	Central Nervous System
DD	Drowsiness Detection
DL	Deep Learning
DA	Data Augmentation
DSNs	Deep Stacking Networks
DE	Differential Entropy
DRL	Driven Right Leg
EEG	Electroencephalogram
ECG	Electrocardiogram
EMG	Electromyogram
EOG	Electrooculogram
EDF	European Data Interface
FIR	Finite Impulse Response
FFT	Fast Fourier Transformation
FP	False Positive
FN	False Negative
GPU	Graphics Processing Unit
IIR	Infinite Impulse Response
LSTM	Long Short Term Memory
ML	Machine Learning
NIRS	Near Infrared Spectroscopy
PERCLOS	Percentage of eye closure
RNNs	Recurrent Neural Networks
ResNet	Residual Network
RMSprop	Root Mean Squence Propagation
ReLU	Rectified Linear Unit
SGD	Stochastic Gradient Descent Optimizer
TP	True Positive
TN	True Negative
VGGNet	Visual Geometry Group Network
